# Association between social psychological status and efavirenz and nevirapine plasma concentration among HIV patients in Kenya

**DOI:** 10.1038/s41598-021-01345-9

**Published:** 2021-11-11

**Authors:** Musa Otieno Ngayo, Margaret Oluka, Wallace Dimbuson Bulimo, Faith Apolot Okalebo

**Affiliations:** 1 Centre of Microbiology Research, Kenya Medical Research Institute, Complex off Ngong Road Box, Nairobi, 19464-00202 Kenya; 2 Department of Pharmacology and Pharmacognosy, University of Nairobi, Nairobi, Kenya; 3 Department of Biochemistry, University of Nairobi, Nairobi, Kenya

**Keywords:** Psychology, Health care, Risk factors

## Abstract

HIV-related stigma, lack of disclosure and social support are still hindrances to HIV testing, care, and prevention. We assessed the association of these social-psychological statuses with nevirapine (NVP) and efavirenz (EFV) plasma concentrations among HIV patients in Kenya. Blood samples were obtained from 254 and 312 consenting HIV patients on NVP- and EFV-based first-line antiretroviral therapy (ART), respectively, and a detailed structured questionnaire was administered. The ARV plasma concentration was measured by liquid chromatography-tandem mass spectrometry (LC–MS/MS). There were 68.1% and 65.4% of the patients on NVP and EFV, respectively, who did not feel guilty for being HIV positive. The disclosure rates were approximately 96.1% and 94.6% of patients on NVP and EFV, respectively. Approximately 85% and 78.2% of patients on NVP and EFV, respectively, received social support as much as needed. There were 54.3% and 14.2% compared to 31.7% and 4.5% patients on NVP and EFV, respectively, with supratherapeutic and suboptimal plasma concentrations. Multivariate quantile regression analysis showed that feeling guilty for being HIV positive was associated with increased 954 ng/mL NVP plasma concentrations (95% CI 192.7 to 2156.6; *p* = 0.014) but not associated with EFV plasma concentrations (adjusted *β* = 347.7, 95% CI = − 153.4 to 848.7*; p* = 0.173). Feeling worthless for being HIV positive was associated with increased NVP plasma concentrations (adjusted *β* = 852*,* 95% CI = 64.3 to 1639.7*; p* = 0.034) and not with EFV plasma concentrations (adjusted *β* = − 143.3*,* 95% CI = − 759.2 to 472.5*; p* = 0.647). Being certain of telling the primary sexual partner about HIV-positive status was associated with increased EFV plasma concentrations (adjusted *β* 363, 95% CI, 97.9 to 628.1; *p* = 0.007) but not with NVP plasma concentrations (adjusted *β* = 341.5*,* 95% CI = − 1357 to 2040*; p* = 0.692). Disclosing HIV status to neighbors was associated with increased NVP plasma concentrations (adjusted *β* = 1731*,* 95% CI = 376 to 3086*; p* = 0.012) but not with EFV plasma concentrations (adjusted *β* = − 251*,* 95% CI = − 1714.1 to 1212.1*; p* = 0.736). Obtaining transportation to the hospital whenever needed was associated with a reduction in NVP plasma concentrations (adjusted *β* = − *1143.3,* 95% CI = − 1914.3 to − 372.4*; p* = 0.004) but not with EFV plasma concentrations (adjusted *β* = − 6.6*,* 95% CI = − 377.8 to 364.7*; p* = 0.972). HIV stigma, lack disclosure and inadequate social support are still experienced by HIV-infected patients in Kenya. A significant proportion of patients receiving the NVP-based regimen had supra- and subtherapeutic plasma concentrations compared to EFV. Social-psychological factors negatively impact adherence and are associated with increased NVP plasma concentration compared to EFV.

## Introduction

Although the current trend in the global HIV epidemic has stabilized, data still imply disappointingly high levels of infection, an indictment of irregular control progress in countless countries^1^. The HIV pandemic continues to be the leading cause of death in sub-Saharan Africa, with Kenya having the joint third-largest HIV epidemic in the world (alongside Tanzania), with 1.6 million people living with HIV^1^. HIV infection affects every breadth of life, including physical, psychological, social and spiritual dimensions^2,3^. In as much as HIV infection has been reported in Kenya for the last four decades, this infection is still dreaded by many, mainly due to misinformation about the disease and consequently the stigma and exclusion associated with the infection^4^. People living with HIV (PLWHA) are burdened with both medical and social problems associated with the disease^5^. HIV infection among a large population results in stigma for both infected and affected individuals^6,7^. Furthermore, infection consistently results in loss of socioeconomic status, employment, income, housing, health care and mobility^5^. The outcome of stigma includes but is not limited to increased secrecy (lack of disclosure) and denial, which is not only a stimulus for HIV transmission but also a cause for poor disclosure and subsequent lack or inadequate social support^5,7^.

Antiretroviral therapy (ART) is an integral component in reducing the burden of HIV. Globally, at the end of 2020, 67% of 38 million PLWHA were on ART^1^. A remarkable scale-up of ART has put Kenya on track to reach the target for AIDS-related deaths. At the end of 2020, approximately 74% of adults and 73% of children in Kenya needing ART were essentially receiving it^1^. A remarkable fraction of these patients (68%) had attained viral suppression (UNAIDS, 2020). At the time of this study, the first-line ART guidelines for children, youth and adults in Kenya typically contained a backbone of two nucleoside reverse transcriptase inhibitors (NRTIs; zidovudine [AZT], or tenofovir [TDF] with lamivudine [3TC]), plus one nonnucleoside reverse transcriptase inhibitor (NNRTI), either nevirapine (NVP) or efavirenz (EFV)^8^.

Therapeutic drug exposure is a major requirement for ART management^9^. Suboptimal exposure to ART, especially NNRTIs (NVP and EFV), jeopardizes ART treatment success^10^. Generally, efavirenz and nevirapine plasma concentrations are associated with several factors, including host pharmacogenetics, as well as pharmacoecological factors, such as social-psychological status and adherence^11^. Although pharmacoecological factors are those that primarily affect adherence, social psychological status could independently affect ARV plasma concentration^11^. HIV stigma negatively affects ART utilization and the quality of care^5^. Social support and disclosure have been shown to significantly affect treatment outcomes in many settings^4^. Counselling and social support for both infected and affected people is associated with effective coping with each stage of the infection and enriches the quality of life and hence adherence to ART^2^. This study assessed the association between HIV stigma, disclosure and social support on ART adherence and the steady-state plasma concentrations of NVP and EFV among HIV patients receiving ART in one of the largest and oldest cosmopolitan care and treatment centers in Kenya.

## Methods

### Study design and setting

This was a cross-sectional study conducted between August 2016 and January 2020. The data presented in this study were part of a study that aimed to assess the pharmacogenetic and pharmacoecological etiology of suboptimal responses to nonnucleoside reverse transcriptase inhibitors (NNRTIs) among HIV patients in Nairobi, Kenya. Patients were recruited in this study if they were HIV-infected adults (aged above 18 years); receiving first-line ART comprising zidovudine (AZT) or abacavir (ABC) or tenofovir (TDF) or stavudine (d4T), lamivudine (3TC), and efavirenz (EFV) or nevirapine (NVP) for at least 12 months; and willing to give voluntary written informed consent. This study was based at the Family AIDS Care and Educational Services (FACES) based at Kenya Medical Research Institute (KEMRI) in Nairobi Kenya. The ART regimen formulation and dosing used in this study were performed according to the guidelines of the Ministry of Health, National AIDS & STI Control Program^8^. The EFV-based ART regimen comprised the following: ABC 300 mg/3TC 150 mg combination taken twice daily plus EFV 600 mg once daily, TDF 300 mg/3TC 300 mg/EFV 600 mg one fixed dose combination taken once daily, or AZT 300 mg/3TC 150 mg combination taken twice daily plus EFV 600 mg once daily. The NVP based regimen comprised the following: ABC 600 mg/3TC 300 mg combination taken once daily plus NVP 200 mg twice daily, or TDF 300 mg/3TC 300 mg combination taken once daily plus NVP 200 mg twice daily, or AZT 300 mg /3TC 150 mg NVP 200 mg one fixed dose combination taken twice daily and D4T 30 mg/3TC 150 mg/NVP 200 mg one fixed dose combination taken twice daily. The study population and site have been described in detail in our previous publication Ngayo et al.^12^. This research was carried out in accordance with the basic principles defined in the Guidance for Good Clinical Practice and the Principles enunciated in the Declaration of Helsinki (Edinburg, October 2000). This protocol and the corresponding informed consent forms used in this study were reviewed, and permission was obtained from the Kenya Medical Research Institute Scientific Review Unit (SERU) (Protocol No SSC 2539). Written informed consent was obtained from all patients before enrollment.

### Sample size

Sample size calculation used the formula described by Lemashow^13^ based on population proportion estimation with specified relative precision. The alpha (α) was set at 0.05, the relative precision (ε) was set at 0.20 and the proportion of HIV-infected individuals with suboptimal NVP/EFV plasma concentrations during a 12-month ART was set at 15%^14,15^. A total of 599 patients were recruited to achieve 0.95 power, where recruitment of patients per treatment arm was done proportionate to size, yielding 269 and 330 patients on NVP- and EFV-based regimens, respectively.

### Data collection

#### ART drug adherence assessment

Screening for adherence to ART in this study was conducted by review of pharmacy refill data or medical records as described by Ochieng et al.^16^. Adherence was measured based on dose compliance during the 30 days preceding the latest refill. The quantity of dose pills at refill was counted and reconciled against the dose counts dispensed at last refill. Furthermore, pill count data were obtained from patient cards for the four months preceding the study period. Nonadherence was determined as the percentage of overdue dose at refill, averaged over a four-month period and used to assign adherence as good (< = 5% dose skipped), fair (6–15% dose skipped) or poor (> 15% dose skipped).

#### Structured interviews

Structured interviews (Supplementary file) were used to collect patient-related information from all the study patients. The data collected included demographic characteristics, clinical history, HIV stigma, HIV disclosure and social support and adherence. A pilot study was conducted to test the questionnaire and other key points in the interviews. Some of the key points explored in the structured questionnaire included stigma and segregation of people living with HIV (self-worth, guilt, emotional feeling); challenges of living with HIV, such as access to health services and community life; experiences/issues with HIV disclosure and adherence to medications. The interviews were conducted by a clinician in a separated private room. The second part of the questionnaire was filled out by retrospective review of patient medical records to abstract data on the occurrence of any adverse drug reactions, evidence of treatment failures and adherence to ART.

Whole blood samples (5 mL) at 12–16 h post ARV drug dose were collected using EDTA anticoagulant tubes to determine the concentration of NVP and EFV plasma concentrations.

#### Determination of nevirapine and efavirenz plasma concentrations

The nevirapine and efavirenz plasma concentrations were measured using a tandem quadrupole mass spectrometer (LC/MS/MS) designed for ultrahigh performance: Xevo TQ-S (Waters Corporation, U.S. A) as described by Reddy et al*.*^17^. Plasma samples were first subjected to a thorough in-house method for the inactivation of the HIV virus. Plasma samples were extracted using Bond Elut C18 cartridges according to the manufacturer’s instructions (Agilent Technologies, USA). The eluents were then completely evaporated using Thermo Scientific™ Reacti-Vap™ Evaporators (Thermo Fisher Scientific Inc., USA) at 37 °C for 30 min. This was then reconstituted using 100μl100 ul of equal parts 1:1 acetonitrile and water, vortexed briefly and transferred into 50 ml capped vials and placed into Xevo TQ-S (Waters Corporation, U.S. A) for quantification. Approximately 1 μl of the samples was injected automatically into the LC/MS/MS instrument and quantified within 5 min.

### Data analysis

All data were subjected to descriptive data analysis. Frequencies and percentages were used to present the sociodemographic data. The relationship between HIV stigma, disclosure and social support-related variables and ART drug adherence was first evaluated using the chi-square test or Fisher’s exact test. The social-psychological variables were then analyzed for association with NVP and EFV plasma concentrations. Steady-state NVP and EFV plasma concentrations were not normally distributed by the Shapiro–Wilk test; hence, the Kruskal–Wallis test and Dunn’s test and quantile regression analysis were used to evaluate variations and associations with NVP and EFV plasma concentrations at the 5% significance level. All statistical analyses were performed using STATA v 13 (StataCorp LP, Texas, USA). The NVP plasma concentrations were categorized as < 3400 ng/mL (below the therapeutic range), 3400–6000 ng/mL (therapeutic range) and > 6000 ng/mL (above the therapeutic range). For EFV, concentrations of < 1000 ng/ml were considered below the therapeutic range, 1000 to 4000 ng/ml considered the therapeutic range and > 4000 ng/ml considered supratherapeutic concentrations^18,19^.

### Ethics approval

Ethical approval for this study was obtained from the KEMRI Scientific Review Unit (SERU). The protocol number is SSC No. 2539.

### Consent to participate

Written informed consent was obtained from all subjects before the study.

## Results

### Baseline characteristics of study patients

Table 1 summarizes the baseline characteristics of the study population. The results from the 254/269 (94.4%) and 312/330 (94.5%) response rates of patients on NVP and EFV, respectively, with all the relevant data were analyzed. The median age of the patients was 41 years (IQR = 35–47 years), with a median duration of living with HIV infection of five years (IQR = 1–11 years) and a median duration since ART initiation of three years (IQR = 1–8 years). Among these patients, 342 (60.4%) were female, 379 (67%) were married, 367 (64.8%) were Bantus, and 106 (18.2%) had a previous partner who died. Only 3.5% and 5.8% and 19.7% and 17.3% (on NVP and EFV, respectively) were currently smoking and taking alcohol, respectively.

**Table 1:** Baseline characteristics of the study patients.

Variable		All patients (n = 566)		Nevirapine (n = 254)		Efavirenze (n = 312)		*p value*
		n	(%)	n	(%)	n	(%)	
Age (years)	**Median (IQR)**	41	(35–47)	42	(36–48)	40	(34–47)	0.046
	20–30	66	11.7	25	9.8	41	13.1	
	31–40	210	37.1	84	33	126	40.4	
	41–50	202	35.7	106	41.7	96	30.8	
	> 51	88	15.5	39	15.4	49	15.7	
Gender	Female	342	60.4	163	64.2	179	57.4	0.102
	Male	224	39.6	91	35.8	133	42.6	
Marrital status	Married	379	67	165	65.0	214	68.6	0.703
	Single	154	27.2	72	28.4	82	26.3	
	Divorced	26	4.6	14	5.5	12	3.9	
	Widow	7	1.2	3	1.2	4	1.3	
Occupation	Employed	193	34.1	80	31.5	113	36.2	0.354
	Unemployed	102	18	44	17.3	58	18.9	
	Self employed	271	47.9	130	51.2	141	45.2	
Ethnicity	Bantu	367	64.8	161	63.4	206	66.0	0.256
	Nilotes	190	33.6	91	35.8	99	31.7	
	Cushites	9	1.7	2	0.8	7	2.2	
Education level	Primary	174	30.7	69	27.2	105	33.4	0.17
	Secondary	203	35.9	102	40.2	101	32.4	
	Tertiary	182	32.2	81	31.9	101	32.4	
	Non-formal	7	1.2	2	0.8	5	1.6	
Cigarette smoking	Yes	27	4.8	9	3.5	18	5.8	0.24
	No	539	95.2	245	96.5	294	94.3	
Alcohol consumption	Yes	104	18.4	50	19.7	54	17.3	0.099
	No	462	81.6	204	80.3	258	82.7	
Age of sexual debut (Years)	**Median (IQR)**	18	(17–20)	18	(17–19)	18	(17–20)	0.929
	< 18	371	65.6	166	65.4	205	65.7	
	> 18	195	34.5	88	34.7	107	34.3	
Lifetime sexual partners	**Median (IQR)**	2	(1–5)	2	(1–4)	3	(1–5)	0.019
	None	3	0.5	2	0.8	1	0.3	
	1	214	37.8	110	43.3	104	33.3	
	> 1	349	61.7	142	55.9	207	66.4	
Current ART regimen	3TC, ABC, EFV	1	0.2	0	0	1	0.3	0.0001
	3TC, TDF, EFV	187	33.1	0	0	187	59.9	
	3TC, ZDV, EFV	124	21.9	0	0	124	39.7	
	3TC, ABC, NVP	1	0.2	1	0.4	0	0	
	3TC, TDF, NVP	159	28.1	159	62.6	0	0	
	3TC, ZDV, NVP	93	16.4	93	36.6	0	0	
	3TC, d4T, NVP	1	0.2	1	0.4	0	0	
Difficult to tell others about my HIV infection	Agree	418	73.8	189	74.4	229	73.4	0.848
	Disagree	148	26.2	65	25.6	883	26.6	
Feeling guilty for being HIV positive	Agree	189	33.4	81	31.9	108	34.6	0.531
	Disagree	377	66.6	173	68.1	204	65.4	
Feeling worthless for being HIV positive	Agree	137	24.2	55	21.7	82	26.3	0.236
	Disagree	429	75.8	199	78.4	230	73.7	
Hide HIV status from others	Agree	403	71.2	186	73.2	217	69.5	9.352
	Disagree	163	28.8	68	26.8	95	30.5	
Disclose HIV status to anyone	Yes	539	95.2	244	96.1	295	94.6	0.435
	No	27	4.7	10	3.9	17	5.4	
Disclosed HIV status to partner or spouse	Yes	446	78.8	204	80.3	242	77.8	0.665
	No	63	11.1	25	9.8	38	12.2	
	Not applicable	57	10.1	25	9.8	32	10.3	
Disclosed HIV status to family members	Yes	349	61.7	166	65.4	183	58.7	0.178
	No	212	37.5	87	34.4	125	40.1	
	Not applicable	5	0.9	1	0.4	4	1.3	
Disclosed HIV status to the public	Yes	12	2.1	5	1.9	7	2.2	0.965
	No	513	90.6	231	90.4	282	90.4	
	Not applicable	41	7.2	18	7.1	23	7.4	
Get useful advice about important things in life	As much as I wouild like	460	81.3	216	85.0	244	78.2	0.022
	Less than I wouild like	79	13.9	33	12.9	46	14.7	
	Much less than I wouild like	11	1.9	1	0.4	10	3.2	
	Never	16	2.8	4	1.6	12	3.9	
Get financial help during emergency	As much as I wouild like	337	59.5	162	63.8	175	56.1	0.066
	Less than I wouild like	92	16.3	40	15.8	52	16.7	
	Much less than I wouild like	44	7.8	12	4.7	32	10.3	
	Never	93	16.4	40	15.8	53	16.9	
Get transportation help when needed	As much as I wouild like	357	63.1	169	66.5	188	60.3	0.19
	Less than I wouild like	81	14.3	38	14.9	43	13.8	
	Much less than I wouild like	45	7.9	15	5.9	30	9.6	
	Never	83	14.7	32	12.6	51	16.4	
Get general help when sick	As much as I wouild like	456	80.6	212	83.5	244	78.2	0.437
	Less than I wouild like	67	11.8	27	10.6	40	12.8	
	Much less than I wouild like	18	3.2	6	2.4	12	3.9	
	Never	25	4.4	9	3.5	16	5.2	

Out of 254 patients on NVP and 312 on EFV, the majority 74.4% and 73.3% stated difficulties disclosing their HIV status. In contrast, the majority (79.1% and 75.9%; 68.1% and 65.4% on NVP and EFV, respectively) did not feel immoral or guilty for being HIV positive, respectively. For patients on either NVP or EFV, the majority did not feel ashamed or worthless for being HIV positive and were very ready to tell their primary sexual partner of their HIV status. The majority, 85% (NVP) and 78.2% (EFV), were satisfied with advice received about important things in life (p = 0.022). Similarly, the majority of these patients had adequate psychosocial support in finding someone to talk to about work/household problems, about personal/family problems and had people who cared about their situations and received much love and affection. The majority of the patients also received emergency financial and transportation support, but there was no significant difference between the ART regimens.

### ART adherence

**Figure 1:**
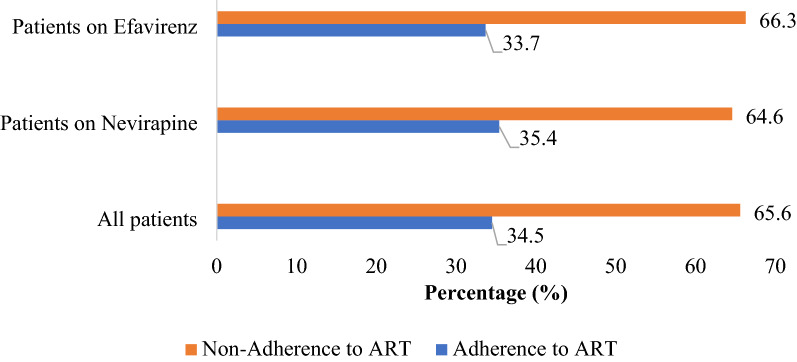
Distribution of patients with ART adherence in the past 30 days.

Among all the study patients, 371 (n = 566; 65.6%), 164 (n = 254; 64.6%) on NVP and 207 (n = 312; 66.3%) on EFV were categorized as poor adherence to ART (Fig. 1).

### Efavirenz and Nevirapine plasma concentration

Among the patients on the nevirapine-based ART regimen, the majority 138 (n = 254; 54.3%) had plasma concentrations of > 6000 ng/ml, which are considered levels for durable viral suppression. There were 80 (n = 254; 31.5%) patients with NVP concentrations between 3400 and 6000 ng/ml considered levels for viral mutant selection windows and a few 3 (n = 254; 14.2%) who had NVP plasma concentrations of < 3400 ng/ml considered levels for poor viral suppression (p = 0.0001). For patients on the efavirenz-based ART regimen, the majority 199 (n = 312; 63.8%) had plasma concentrations between 1000 and 4000 ng/ml considered levels for viral mutant selection windows followed by 99 (n = 312; 31.7%) with EFV plasma concentrations of > 4000 ng/ml considered levels for durable viral suppression. Fourteen (n = 312; 4.5%) patients had EFV plasma concentrations of < 1000 ng/ml, which are considered concentrations for a poor viral suppression window (p < 0.05).

There was no significant difference in NVP plasma concentrations across dosing formulations (p = 0.248) or among EFV dosing formulations (p = 0.352) (Table 2).

**Table 2:** Relationship between HIV stigma, disclosure and social support and ART drug adherence.

Variable	FISHER’S EXACT TEST	
	ART drug Adherence	
	Nevirapine	Efavirenz
	*P value*	*P value*
**Socio-demographic variables**		
Gender	0.318	0.253
Age	0.393	0.129
Marital status	0.06	0.368
Occupation	0.952	0.565
Religion	0.785	0.689
Education	0.611	0.124
Vacational schooling	0.482	0.209
Living with partner	0.363	0.871
Had more than one partner	0.97	0.533
Previous partner died	0.919	0.953
Smoking	0.725	0.798
**HIV stigma related factors**		
Difficult to tell others about my HIV infection	0.234	0.281
Being HIV positive makes me feel immoral	0.260	0.005
Being HIV positive makes me feel guilty	0.035	0.314
Being HIV positive makes me feel ashamed	0.570	0.794
Being HIV positive makes me feel it worthless	0.750	0.344
Being HIV positive makes me feel it is my own fault	0.111	0.318
Hide HIV status from others	0.005	0.605
Feel certain to tell primary sexual partner being HIV positive	0.0001	0.0001
**HIV disclosure related factors**		
Disclose HIV status to anyone	0.332	0.033
Disclosed HIV status to partner or spouse	0.197	0.578
Disclosed HIV status to family members	0.570	0.730
Disclosed HIV status to friends	0.908	0.383
Disclosed HIV status to neighbor	0.306	0.202
Disclosed HIV status to employers	0.217	0.579
Disclosed HIV status to religious leaders	0.362	0.582
Disclosed HIV status to the public	0.748	0.331
Number disclosed about HIV status in the family	0.185	0.055
**HIV social support**		
Get useful advice about important things in life	0.022	0.005
Get chance to talk to someone about work or household problems	0.005	0.001
Get chance to talk to someone about personal or family problems	0.071	0.002
I have people who cares about what happens to me	0.256	0.038
I get love and affection	0.0001	0.008
Help with household duties	0.007	0.001
Get financial help during emergency	0.005	0.045
Get transportation help when needed	0.001	0.014
Get general help when sick	0.138	0.009

### Relationship between HIV-related stigma, disclosure and social support and ART adherence

The HIV stigma-related factors associated with adherence to NVP-based regimens included feeling guilty for being HIV positive, hiding HIV status from others and feeling certain to tell primary sexual partners about HIV status. Feeling immoral for being HIV positive and feeling certain to tell primary sexual partners about HIV status was associated with adherence to EFV-based regimens.

Being able to disclose HIV status to anyone and to family members was associated with adherence to EFV-based regimens. The majority of HIV social support-related factors, including getting useful advice about important things in life, getting a chance to talk to someone about work or household problems, getting love and affection, was associated with ART adherence to both NVP- and EFV-based regimens (Table 2).

### Variation in median nevirapine and efavirenz plasma concentrations and HIV stigma, disclosure and social support-related factors

Table 3 summarizes the variation in the median NVP and EFV plasma concentrations and sociodemographic, sexual behavior, HIV stigma and disclosure characteristics. Patients who disclosed their HIV status to their employer had higher median (IQR) EFV plasma concentrations (3157, IQR = 2001–5976 ng/mL) than those who did not (2173.5, IQR = 1655.5–3208.5 ng/mL; *p* = 0.041). Patients who did not disclose their HIV status to religious leaders had higher median (IQR) EFV plasma concentrations (2821.5, IQR = 1945–5270 ng/mL) than those who did (1998.5, IQR = 1548–2520 ng/mL; *p* = 0.0031). Furthermore, patients who disclosed their HIV status to the public had higher median (IQR) EFV plasma concentrations (3097, IQR = 2872–5976 ng/mL) than patients who did not (1965, IQR = 1639–2763 ng/mL; *p* = 0.0117).

**Table 3:** Variation in median nevirapine and efavirenz plasma concentration and HIV stigma, disclosure and social support-related variables.

Variable	NEVIRAPINE (N = 254)					EFAVIRENZ (N = 312)				
	n	Median	(IQR)		P	n	Median	(IQR)		P
**Age group (Years)**										
20–30	25	6034	4448	7817		41	2961	1679	4603	
31–40	84	6207	4558	8946.5	0.667	126	2698.5	1918	5976	0.476
41–50	106	6368	4599	9784		96	2685.5	1950.5	4282.5	
> 51	39	6011	4518	8843		49	2754	1833	4074	
**Gender**										
Male	91	5917	4449	8638	0.387	133	2747	1918	5336	0.728
Female	163	6364	4558	9293		179	2712	1868	4647	
**HIV drug resistant mutation**										
Yes	24	6062	4119	8786	0.519	10	1373.5	49	2807	0.006
No	230	6237.5	4532	9163		302	2758.5	1918	5139	
**HIV viral load (Cells/mls)**										
< 1000	230	6237.5	4532	9095	0.609	300	2764	1919.5	5171.5	0.002
≥ 1001	24	6062	4119	8866		12	1373.5	52.5	2714	
**Current ART regimen**										
3TC, ABC	1	8798	8798	8798		1	1434	1434	1434	
3TC, TDF	159	6698	4599	9755	0.2481	187	2621	1838	5139	0.3519
3TC, ZDV	93	5729	4448	8323		124	2796.5	1968	4726.5	
3TC, d4T	1	3552	3552	3552						
**Being HIV positive makes me feel guilty**										
Agree	81	5557	4247	7633	0.016	108	2645.5	1895	5171.5	0.927
Disagree	173	6511	4607	9863		204	2854	1869	4839.5	
**Being HIV positive makes me feel it worthless**										
Agree	55	5243	3975	7311	0.054	82	2756	1951	4319	0.837
Disagree	199	6511	4599	9755		230	2720.5	1838	5204	
**Disclosed HIV status to partner or spouse**										
Yes	204	6402.5	4564.5	9180.5		242	2759.5	1886	5204	
No	25	4853	3450	6202	0.036	38	2991	1918	5336	0.565
Not applicable	25	6273	4577	9909		32	2556	1750	3488	
**Disclosed HIV status to family members**										
Yes	166	5967.5	4444	7966		183	2592	1917	5044	
No	87	6868	4951	10,635	0.064	125	2867	1870	4911	0.312
Not applicable	1	8034	8034	8034		4	1699.5	456.5	3118.5	
**Disclosed HIV status to neighbor**										
Yes	13	5239	3631	7009		22	3079	1917	7572	
No	234	6237.5	4558	9095	0.210	280	2739.5	1902	4837	0.088
Not applicable	7	7966	6372	9909		10	2027.5	857	2961	
**Disclosed HIV status to religious leaders**										
Yes	18	4479	2960	7009		26	2440	1633	5909	
No	222	6317	4607	9293	0.055	264	2821.5	1945	5270	0.003
Not applicable	14	6371.5	4211	8034		22	1998.5	1548	2520	
**Disclosed HIV status to public**										
Yes	5	5736	5239	7009		7	3097	2872	5976	
No	231	6202	4503	9163	0.869	282	2766.5	1918	5139	0.012
Not applicable	18	6595.5	4558	8034		23	1965	1639	2763	
**Get financial help during emergency**										
As much as I wouild like	162	6365.5	4558	8964		175	2836	1918	4911	
Less than I wouild like	40	5468.5	4275	8191.5	0.492	52	2309.5	1789.5	5038	0.797
Much less than I wouild like	12	7275.5	6056.5	9583		32	2747.5	1615.5	8797.5	
Never	40	5710.5	4046	9867.5		53	2872	2043	4241	
**Get transportation help when needed**										
As much as I wouild like	169	6538	4571	9198		188	2821.5	1895	5223.5	
Less than I wouild like	38	5527.5	4336	8382	0.550	43	2462	1818	4872	0.917
Much less than I wouild like	15	6202	4180	6868		30	2670	1679	6875	
Never	32	5635.5	3955.5	8750		51	2786	1942	3875	
**Get general help when sick**										
As much as I wouild like	212	6351	4448.5	9129		244	2796.5	1895	4977.5	
Less than I wouild like	27	5911	4990	9411	0.970	40	2569	1999.5	5589.5	0.534
Much less than I wouild like	6	7039	5729	8405		12	2447.5	911	4693	
Never	9	5692	5457	7009		16	2931.5	1613.5	4168	

Patients with higher median (IQR) EFV plasma concentrations were those who did not feel guilty for being HIV positive (6511, IQR = 4607–9863 ng/mL) compared to patients who felt guilty (5557, IQR = 4247–7633 ng/mL; *p* = 0.0163). Patients who disclosed their HIV status to their spouse (6402.5, IQR = 4564.5–9180.5 ng/mL) had higher median (IQR) NVP plasma concentrations than those who did not (4853, IQR = 3450–6202 ng/mL; *p* = 0.0362).

### Factors associated with drug plasma concentrations

#### Stigma

In multivariate quantile regression analysis, feeling guilty for being HIV positive (adjusted *β* = 954*,* 95% CI = 192.7 to 2156.6*; p* = 0.014) or feeling worthless for being HIV positive (adjusted *β* = 852*,* 95% CI = 64.3 to 1639.7*; p* = 0.034) were independent factors associated with increased NVP plasma concentrations. For patients on EFV, being certain of telling the primary sexual partner about HIV-positive status was associated with increased EFV plasma concentrations (adjusted *β* 363, 95% CI, 97.9 to 628.1; *p* = 0.007) (Table 4).

**Table 4:** Regression analysis between nevirapine and efavirenz plasma concentrations and HIV stigma variables.

Variable	NEVIRAPINE (N = 254)				EFAVIRENZ (N = 312)			
	Unadjusted β	(95% CI)		*p value*	Unadjusted β	(95% CI)		*p value*
Age	− 14	− 56.2	28.2	0.307	− 13.7	− 38.7	11.4	0.284
Gender	447	− 545.5	1439.5	0.376	− 35	− 536.5	466.5	0.891
Alcohol use number of times	− 198	− 680.7	284.7	0.42	330	− 534.4	1194.4	0.453
Age of sexual debut	− 364	− 1385.8	657.8	0.484	54	− 459.7	567.7	0.836
Number of sexual life partners	− 600	− 1285.7	85.7	0.086	− 557	− 918.0	− 196.0	0.003
Number of sexual acts in the past 3 months	− 46.5	− 748.9	655.9	0.896	− 106	− 648.9	436.9	0.701
Presence of HIV drug resistant mutation	− 117	− 2064.4	1830.4	0.906	1388	484.1	2291.9	0.003
Viral load (Cells/mls)	117	− 2474	2708	0.929	− 1390	− 2642.7	− 137.3	0.03
Difficult to tell others about my HIV infection	141	− 958.8	1240.8	0.801	− 126	− 703.9	451.9	0.668
Being HIV positive makes me feel guilty	954	26.7	1881.3	0.044	210	− 281.3	701.3	0.401
Being HIV positive makes me feel it worthless	1268	379.4	2156.6	0.005	− 33	-744.7	678.7	0.927
Feel certain to tell primary sexual partner being HIV positive	372	− 453.2	1197.2	0.376	426	24.3	827.7	0.038
Disclose HIV status to anyone	− 539	− 1578.8	500.8	0.308	983	− 1058.0	3024.0	0.344
Disclosed HIV status to family members	1051.5	− 541.5	2644.5	0.195	134	− 381.8	649.8	0.61
Disclosed HIV status to neighbor	1675	137.5	3212.5	0.033	− 445	− 1441.0	551.0	0.38
Disclosed HIV status to employers	− 112	− 1203.3	979.3	0.84	− 489	− 1037.2	59.2	0.08
Disclosed HIV status to religious leaders	1609	− 98.7	3316.7	0.065	− 410	− 907.9	87.9	0.106
Get useful advice about importat things in life	− 539	− 1778.4	1303.7	0.762	− 134.3	− 483.8	215.1	0.45
Get financial help during emergency	− 124.7	− 541.9	292.6	0.557	18.7	− 189.8	227.1	0.86
Get transportation help when needed	− 300	− 512.1	− 87.9	0.006	− 5	− 177.6	167.6	0.955
Get general help when sick	− 217	− 599.7	165.7	0.265	− 158	− 562.3	246.3	0.442

Variable	NEVIRAPINE (N = 254)				EFAVIRENZ (N = 312)			
	Adjusted β	(95% CI)		*p value*	Adjusted β	(95% CI)		*p value*
Age	0.421	− 71.7	72.5	0.991	− 15.5	− 52.5	21.6	0.412
Gender	172	− 1010.5	1354.5	0.775	− 40.4	− 832.7	751.9	0.92
Alcohol use number of times	− 162.5	− 811	486	0.622	398	− 431.2	1227.2	0.346
Age of sexual debut	− 1008.1	− 2745.4	729.1	0.254	563.5	− 424.6	1551.6	0.263
Number of sexual life partners	− 988	− 2156.8	180.8	0.097	− 845.7	− 1315.0	− 376.4	0.0001
Number of sexual acts in the past 3 months	− 2180.8	− 5358.2	996.6	0.178	487.3	− 3224.2	4198.8	0.796
Presence of HIV drug resistant mutation	226.1	− 7513.4	7965.6	0.954	− 1192.0	− 5251.2	2867.2	0.564
Viral load (Cells/mls)	559.9	− 6645.1	7764.9	0.878	0.0	0.0	0.1	0.339
Difficult to tell others about my HIV infection	− 528.5	− 1633.9	576.9	0.347	− 177	− 1021.3	667.3	0.68
Being HIV positve makes me feel guilty	954	192.7	1715.3	0.014	347.7	− 153.4	848.7	0.173
Being HIV positve makes me feel it worthless	852	64.3	1639.7	0.034	− 143.3	− 759.2	472.5	0.647
Feel certain to tell primary sexual partner being HIV positive	341.5	− 1357.0	2040.0	0.692	363	97.9	628.1	0.007
Disclose HIV status to anyone	− 1042.9	− 2597.4	511.6	0.188	1342	1653.6	4337.6	0.379
Disclosed HIV status to family members	812.9	− 483.3	2109.1	0.218	245	− 365.8	855.8	0.431
Disclosed HIV status to neighbor	1731	376.0	3086.0	0.012	− 251	− 1714.1	1212.1	0.736
Disclosed HIV status to employers	− 393.5	− 1586.1	799.1	0.516	− 505	− 1410.3	400.3	0.273
Disclosed HIV status to religious leaders	241.6	− 1675.6	2158.7	0.804	29	− 1120.3	1178.3	0.96
Get useful advice about importat things in life	− 112.7	− 1430.0	1204.6	0.866	16.4	− 400.5	433.4	0.938
Help with household duties	− 315.2	− 1460.0	829.6	0.588	− 226.4	− 556.1	103.4	0.178
Get financial help during emergency	779.3	− 291.9	1850.6	0.153	245.0	− 304.7	794.7	0.381
Get transportation help when needed	− 1143.3	− 1914.3	− 372.4	0.004	− 6.6	− 377.8	364.7	0.972
Get general help when sick	212.3	− 560.5	985.1	0.589	74.1	− 478.3	626.5	0.792

#### Disclosure

In multivariate quantile regression analysis, disclosing patients’ HIV status to neighbors (adjusted *β* = 1731*,* 95% CI = 376 to 3086*; p* = 0.012) was associated with increased NVP plasma concentrations. None of the HIV disclosure-related factors were associated with EFV plasma concentrations (Table 4).

#### Social support

In multivariate quantile regression analysis, transportation to the hospital whenever needed (adjusted *β* = − *1143.3,* 95% CI = − 1914.3 to − 372.4*; p* = 0.004) was associated with lower NVP plasma concentrations. None of the HIV social support-related factors were found to be associated with EFV plasma concentrations (Table 4).

## Discussion

Every blueprint and policies geared towards individualization of ART treatment aimed at prolonging the life of HIV patients contributes significantly to the components of HIV treatment programs in many countries, including Kenya. The recommendation by the World Health Organization (WHO) requiring testing and treatment of all HIV-positive patients regardless of their CD4 or viral load^20^ must also appreciate that optimal ART outcomes require an in-depth understanding of the individual’s variation in response to ART, both efficacy and toxicity. The concentration of ARV drug found in plasma has been shown to affect the rate at which ARVs begin to suppress viral replication and/or the duration of the effect on viral replication^21^. Therapeutic drug concentrations are therefore a key to successful ART^14^. Low drug concentrations observed in patients on ART are related to failure to achieve immediate virologic success and longer-term immunological failure^22^. ARV drug plasma concentrations are associated not only with patients’ pharmacogenetic and pharmacoecological factors^23^ but also to social psychological (defined as human behavior as a result of the relation between mental state and social situation) well-being of patients. Stigma, disclosure and social support are social psychological—mental representations are important influence of our interactions with others and environment. This is among the first studies to assess the association between HIV stigma (a mark of disgrace, discounting, discrediting and discriminating associated with HIV infection and ARV use)^24^, HIV disclosure (action of making new or secret of being HIV positive known) and HIV social support (the perception and actuality that one is cared for or having assistance available from other people) on the steady-state plasma concentrations of nevirapine and efavirenz among HIV patients receiving treatment in Nairobi Kenya.

HIV stigma, disclosure and availability of social support are key determinants of patients’ behavior and are associated with adherence to HIV care, treatment and prevention. Previously, in Kenya, involvement in community support networks considerably enriched adherence and treatment outcome^25^. Furthermore, patients vigorously partaking in community support networks tended to attain peak NVP plasma concentrations early hours postdosing, which were markedly higher than those seen in patients not actively involved in community support networks. Countless studies have interconnected social support to better medication adherence and better clinical outcomes^26^.

The association of patients’ social psychological status with ARV plasma concentration and treatment outcomes might be multifactorial. Social psychological status could indirectly be associated with ARV plasma concentration and treatment outcomes by affecting adherence to ART^25,27,28^. In this study, social psychological factors were significantly associated with adherence among patients on EFV compared to those on NVP. The EFV-based regimen is prescribed as a fixed-dose, single-tablet regimen, while NVP is prescribed as two or more pills per day^8^. It is possible that the higher pill burden among patients on NVP could be associated with the patient’s social psychological status and adherence and hence NVP plasma concentration. Studies have related a lower pill burden with both better adherence and virological suppression^29,30^ as well as patients’ emotional satisfaction^31^. Although not investigated in this study, studies have reported a common cause between social psychological status and non-adherence, both of which could independently be associated with ARV plasma concentration^27,28^. Reverse causality is also possible; efavirenz is associated with high rates of neuropsychiatric side effects, including vivid dreams, insomnia and mood changes, which could impact internal feelings of shame and interest in seeking social support^32^. It is presumed that this neuropsychiatric effect of EFV could affect treatment outcomes, including ARV plasma concentration.

HIV-associated stigma-related factors such as feeling guilty and worthless for being HIV positive were associated with higher median NVP plasma concentrations. For patients on an EFV-based regimen, those who were certain to reveal their HIV status to their primary sexual partner had better ART adherence accompanied by higher median EFV plasma concentrations. Stigma and discrimination remain the paramount challenges confronted by people living with HIV/AIDS^33^. Although data are skewed on the association between HIV stigma and NNRTI plasma concentrations, stigma and discrimination negatively affect people living with HIV^34^. HIV-related stigma is a wide-ranging and worldwide social phenomenon that is exhibited within multiple social spheres, including healthcare encompassing denial of care or treatment, HIV testing without consent, confidentiality breaches, negative attitudes and humiliating practices by health workers^35^. Studies have shown an association between HIV stigma and poorer physical and mental health outcomes^27^. Stigma has also been linked with secondary health-related factors, including seeking healthcare and adherence to antiretroviral therapy and access to and usage of health and social services^27,28^. Inevitably, these negative outcomes of stigma are bound to affect the overall treatment outcomes in terms of therapeutic monitoring.

HIV status disclosure to anyone and family members in this study was associated with ART adherence to an EFV-based regimen and not NVP. In multivariate analysis, disclosure of HIV status to neighbors was associated with increased median NVP plasma concentration. Patients on EFV with lower pill count are more likely to disclose HIV status compared to those on NVP-based regimens, hence better adherence and better treatment outcomes^29,30^. Contrary to our study, in Thailand, Sirikum et al.^36^ reported no significant difference in the median ART adherence by pill count, CD4 count, or HIV viral load between HIV patients who disclosed their status compared to those who did not. Studies have shown that HIV disclosure has two possible treatment outcomes^37^. On the one hand, HIV status disclosure to sexual partners is a vital prevention target underlined by both the WHO and the Centers for Disease Control and Prevention (CDC)^38^. At an individual level and to the general public, HIV disclosure is accompanied by numeral benefits^36^. HIV infection disclosure to sexual partners is associated with less anxiety and increased social support, especially among women^37,38^. Further, HIV status disclosure is accompanied by improved access to HIV prevention and treatment programs, increased opportunities for risk reduction and increased opportunities to plan for the future. Disclosure of HIV status also expands the awareness of HIV risk to untested partners, leading to better acceptance and utilization of voluntary HIV testing and counselling and changes in HIV risk behaviors^37,38^. In addition, disclosure of HIV status to sexual partners empowers couples to make educated reproductive health choices that may eventually lower the number of unintended pregnancies among HIV-positive women^37^. Along with these benefits, however, there are a number of potential risks from disclosure for HIV-infected women, including loss of economic support, blame, abandonment, physical and emotional abuse, discrimination and disruption of family relationships^37,38^. These risks may lead women to choose not to share their HIV test results with their friends, family and sexual partners. This, in turn, leads to lost opportunities for the prevention of new infections and for the ability of patients, especially women, to access appropriate treatment, care and support services where they are available^37,38^.

In our study, patients who had adequate social support, such as getting useful advice about important things in life, having a chance to talk to someone about work, household, personal or family problems, getting love and affection, had higher median NVP and EFZ plasma concentrations. In South Africa, Brittain et al.^39^ showed a correlation between social support and stigma influencing the development of depressive symptoms. The importance of community support networks in enhancing social relationships demystifying HIV-associated stigma is well documented^40,41^. Evidence shows the positive effects of social support and protection on other HIV-related outcomes, such as sexual risk behaviors^42,43^, mental health distress and family relationships^44,45^. Growing evidence of associations between social protection and HIV risk reduction^46^ is reflected in a number of policy documents by UNICEF, UNAIDS and PEPFAR-USAID that focus on pediatric and adolescent HIV prevention^47,48^.

Some of the important limitations worth mentioning in this study included. First, the use of NVP-based ART regimens in Kenya and other countries, especially developed countries, has been considerably reduced in the recent past, meaning that this study could be relevant to a restricted number of patients. Second, standardized tools for measuring stigma, disclosure and social support were not used in this study, limiting the generalizability of this study outcomes. Third, this was a cross-sectional study, which only permitted the description of the relationship between the three sociopsychological factors and NVP/EFV plasma concentrations and not a causal conclusion. Such outcomes can be confirmed in a longitudinal study.

## Conclusions

This study, conducted in one of the oldest and largest cosmopolitan treatment centers in Kenya, shows that HIV stigma, lack of disclosure and inadequate social support are still noticeable among HIV-infected patients in Kenya. The NVP plasma concentrations were highly heterogeneous, with a significant proportion of patients having supratherapeutic and subtherapeutic plasma concentrations compared to those on EFV regimens. Social-psychological factors negatively impact adherence and are associated with increased NVP plasma concentration compared with EFV.

## Supplementary Information

Supplementary Information.

## Data Availability

All data will be stored at figshare at the moment submitted as electronic data.
