# Supplementary material for: Association between social psychological status and efavirenz and nevirapine plasma concentration among HIV patients in Kenya
Source: Sci Rep. 2021 Nov 11;11:22071. doi: 10.1038/s41598-021-01345-9 (PMC8585942; doi:10.1038/s41598-021-01345-9)
Supplement: Supplementary file 1 — Supplementary Information. [file 41598_2021_1345_MOESM1_ESM.pdf]

Influence of social psychological status on efavirenz and nevirapine plasma concentration among HIV patients in Kenya

Musa Otieno Ngayo[1, 2, *], Margaret Oluka[2] Wallace Dimbuson Bulimo[3], Faith Apolot Okalebo[2]

[1]Centre of Microbiology Research, Kenya Medical Research Institute, Nairobi, Kenya; [2]Department of Pharmacology and Pharmacognosy, University of Nairobi, Nairobi, Kenya;

[3]Department of Biochemistry, University of Nairobi, Nairobi, Kenya

* Corresponding author:

Musa Otieno Ngayo; CMR-KEMRI, KNH Complex off Ngong Road Box 19464 – 00202 Nairobi Kenya; Tel: +245(020)2720038; email: musaotieno@yahoo.com or mngayo@kemri.org

STRUCTURED INTERVIEW GUIDE

A. SOCIO-DEMOGRAPHIC

Date of Interview: ☐☐/☐☐/☐☐☐☐ Day Month Year				Participant ID: ☐☐☐☐☐ Interviewer Initials: ☐☐
A.1	What is your age?	☐☐ years		999○Don't know
A.2	What is your date of birth?	☐☐/☐☐/☐☐☐☐ Day Month Year		999○Don't know
A.3	What is your religion?	1○Protestant<br>2○Other Christian<br>3○Seventh Day Adventist	4○Catholic<br>5○Saved/Pentecostal<br>6○Muslim	7○None<br>8○Other __________
A.4	What is the highest level of school you completed?	1○none<br>2○some primary<br>3○primary<br>4○some secondary	5○secondary<br>6○some college<br>7○certificate	8○diploma<br>9○degree<br>10○postgraduate
A.5	Have you completed any other type of vocational school?	1○Yes<br>2○No		
A.6	Have you ever been married?		1○Yes	2○No
A.7	Are you currently married or living together with a partner?		1○Yes	2○No

A.8	Beside you do you have other partner have other wives or does he live with any other women as if married?	1O Yes 2O No
A.9	Do you have a previous husband or partner who died?	1O Yes 2O No
A.11	Did you attend the clinic for antenatal care during your last pregnancy?	1O Yes 2O No
A.12	How many times did you attend antenatal care?	1O 1 time<br>2O 2-3 times<br>3 O 4 or more times
**Now I am going to ask you some questions about transportation.**		
A.13	How long does it normally take to travel from your house to this clinic – a one-way trip?	☐☐☐ **Minutes**<br>Convert all time to minutes. 1 hour = 60 minutes
A.14	How much does it cost for you to travel from your house to this clinic and then back – in other words, round-trip? Include the cost of any overnight stays.	☐ , ☐☐☐ Shillings
Now I am going to ask you about your health and health care in the past 3 months.		
A.15	In the **past 3 months**, were you admitted in a hospital for any reason?	1O yes 2O no

B. ARV HISTORY AND ADHERENCE

B.1	What ARVs are you taking now?	1O LAMIVUDINE, NEVIRAPINE, STAVUDINE<br>2O LAMIVUDINE, NEVIRAPINE, ZIDOVUDINE<br>3O LAMIVUDINE, NEVIRAPINE, TENOFOVIR<br>4O LAMIVUDINE, EFAVIRENZ, TENOFOVIR<br>5O LAMIVUDINE, EFAVIRENZ, ZIDOVUDINE<br>6O LAMIVUDINE, LOPINAVIR, TENOFOVIR, RITONAVIR<br>7O OTHER, Specify________________________
B.2	**Ascertain from the best possible source the following information regarding how ARVs are supposed to be used by participant (i.e., prescribed by health care provider)**	
B 2.1	Prescribed # of times used per day	☐
B.2.2	Prescribed # of pills used at each time	☐
B.2.3	Data obtained from (mark all that apply)	O Pill bottle<br>O Medical document<br>O Subject self-report<br>O Other, specify____________________
B.3	We consider any situation where you stopped taking current ARVs for 30 days or more and when you then re-started it to count as a new episode of use. With this in mind, when did you begin to use current ARVs during your most current episode of use?<br><br>Probe participant to ensure that prior lapses of 30 days or more are counted as beginning of new episode.	☐☐/☐☐/☐☐☐☐<br>Day Month Year<br><br>Leave day or month blank if unknown

B.4	When was the last time you took current ARVs?<br><br>Date should be within 3 days of today's date	☐☐/☐☐/☐☐☐☐<br>Day Month Year<br><br>Leave day or month blank if unknown
B.4.1	Did you ever miss taking current ARVs for a whole day or more?	1 ○ Yes<br>2 ○ No
B.4.2	How many different days did you miss taking current ARVs?	☐☐ days
B.4.3	What was the longest number of days continuously or without a break that you missed taking current ARVs?	☐☐ days
B.4.4	What was the smallest number of days continuously or without a break that you missed taking current ARVs?	☐☐ days
B.5	How many times did you miss taking current ARVs:	
B.5.1	For at least 1 day but no more than 3 days?	☐☐ times
B.5.2	For at least 4 days but no more than 13 days?	☐☐ times
B.5.3	For 14 days or more?	☐☐ times
B.6	Now I want to ask you about the past 3 days. How many doses of current ARVs did you miss:	

	**Days**	**0**	**1**	**2**	**3**
B.6.1	Yesterday?	○	○	○	○
B.6.2	2 days ago?	○	○	○	○
B.6.3	3 days ago?	○	○	○	○

	Refer to the date given in question 3 and record if subject been taking current ARVs for at least the past 30 days.	1 ○ Yes<br>2 ○ No
B.7	In the past 30 days, how many times do you think you missed a dose of *current ARVs*?	☐☐ times
B.8	*Give instrument and pen to participant*<br>Please place an "X" on the line below at the point showing your best guess about how much current ARVs you have taken in the past 30 days<br><br>0% means you have taken no current ARVs, 50% means you have taken half your current ARVs, 100% means you have taken every single dose of current ARVs<br><br>0% 10% 20% 30% 40% 50% 60% 70% 80% 90% 100%	
B.8.1	Other than your current use of current ARVs, was there ever a time in the past when you used this drug?<br><br>Consider breaks of 30 or more days to constitute discrete episodes	1 ○ Yes<br>2 ○ No
B.9	Ask subject and record answers for each episode below. Record the most recent episode first:<br>Consider breaks of 30 days or more to constitute discrete episodes.	

		When did you first start taking this drug?	When did you stop taking this drug?	Calculate length of each episode	Did you take this drug as part of preventing spread

	Prior episode	Start Date mm/yyyy	Stop Date mm/yyyy	Length of Use in months or days.	MTCT of HIV to your baby?
B.9.1	1	☐☐/☐☐☐☐	☐☐/☐☐☐☐	☐☐ 1 O days 2 O months	1 O Yes 2 O No 3 O N/A

B.10	People stop or interrupt taking medications for different reasons. Did you stop/interrupt taking your current ARVs?	1 O Yes<br>2 O ~~No If~~ ▶no, skip to end<br>3 O N/A
B.11	Did you stop or interrupt taking your current ARVs because your doctor wanted you to, you decided to on your own without your doctor's knowledge or you and your doctor came to the decision together?	1 O doctor<br>2 O self<br>3 O together
B.12	I will read a list of possible reasons that caused you to stop/interrupt taking ARVs. Please answer yes or no for each one.	

B.12.1	You didn't have enough money	1 O yes	2 O no	O primary
B.12.2	You were experiencing side effects	1 O yes	2 O no	O primary
B.12.3	You had no transportation to the pharmacy	1 O yes	2 O no	O primary
B.12.4	You felt well or better and felt you didn't need the medications	1 O yes	2 O no	O primary
B.12.5	You felt the medication was not helping you	1 O yes	2 O no	O primary
B.12.6	Your doctor felt the medication was not working	1 O yes	2 O no	O primary

B.12.7	You gave your medications away to someone else	1 O yes	2 O no	O primary
B.12.8	You needed to start medication to treat your TB and therefore could not take your HIV medications	1 O yes	2 O no	O primary
B.12.9	Lapse of drug supply by payment source	1 O yes	2 O no	O primary
B.12.10	You did not have enough food to eat	1 O yes	2 O no	O primary
B.12.11	Were there any other reasons which caused you to stop/interrupt?	1 O yes Specify__________	2 O no	O primary
	If only one reason is cited, then mark that as the primary reason. If more than one reason is cited, ask:<br><br>Please tell me what is the main reason which caused you to stop/interrupt. Mark this as primary reason.			
B.13	If side effects are listed as "yes", ask: I will read a list of possible side effects that may have caused you to stop/interrupt<br>taking ARVs. Please answer yes or no for each one.			

B.13.1	Fatigue or loss of energy	1 O yes	2 O no
B.13.2	Dizziness or lightheadedness	1 O yes	2 O no
B.13.3	Headache	1 O yes	2 O no
B.13.4	Pain, numbness or tingling in the hands or feet	1 O yes	2 O no
B.13.5	Nausea or vomiting	1 O yes	2 O no
B.13.6	Diarrhea or loose bowel movements	1 O yes	2 O no
B.13.7	Bloating, pain or gas in your stomach	1 O yes	2 O no
B.13.8	Problems with weight loss or wasting	1 O yes	2 O no
B.13.9	Problems with fat deposits or weight gain	1 O yes	2 O no
B.13.10	Skin problems, such as rash, dryness or itching	1 O yes	2 O no
B.13.11	Felt nervous or anxious	1 O yes	2 O no

B.13.12	Felt sad, down or depressed	[1]O yes	[2]O no
B.13.13	Was there another side effect you experienced?	[1]O yes<br>Specify__________	[2]O no

C. STIGMA

**Now, please tell me if you either Agree or Disagree with each of these statements**.			
C.1	It is difficult to tell other people about my HIV infection	1 O Agree	2 O Disagree
C.2	Being HIV positive makes me feel immoral.	1 O Agree	2 O Disagree
C.3	I feel guilty that I am HIV positive.	1 O Agree	2 O Disagree
C.4	I am ashamed that I am HIV positive.	1 O Agree	2 O Disagree
C.5	I sometimes feel worthless because I am HIV positive.	1 O Agree	2 O Disagree
C.6	It is my own fault that I am HIV positive	1 O Agree	2 O Disagree
C.7	I hide my HIV status from others	1 O Agree	2 O Disagree
C.8	I feel certain that I can tell my primary sex partner that I have HIV.	1 O Agree	2 O Disagree

D. SOCIAL SUPPORT

Now I am going to ask you some questions about your social supports. For each of these questions, you may answer 'as much as I would like,' "less than I would like", 'much less than I would like', or never.					
**Statement**		**As much as I would like**	**Less than I would like**	**Much less than I would like**	**Never**
D.1	I get useful advice about important things in my life	1○	2○	3○	4○
D.2	I get chances to talk to someone about problems at work or with my housework	1○	2○	3○	4○
D.3	I get chances to talk to someone I trust about my personal and family problems	1○	2○	3○	4○
D.4	I have people who care what happens to me	1○	2○	3○	4○
D.5	I get love and affection	1○	2○	3○	4○
D.6	I get help with household-related work	1○	2○	3○	4○
D.7	I get help with money in an emergency	1○	2○	3○	4○
D.8	I get help when I need transportation	1○	2○	3○	4○
D.9	I get help when I am sick	1○	2○	3○	4○

F. DISCLOUSER

F.1	Have you told anyone about your HIV status?	1O yes	2O no →Skip to next secion	
F.2	Have you told any of the following people about your HIV status?			
F.2.1	Husband/Wife/Partner	1O yes	2O no	3O Not applicable
F.2.2	Other family member(s)	1O yes	2O no	3O Not applicable
F.2.3	Friend	1O yes	2O no	3O Not applicable
F.2.4	Neighbor	1O yes	2O no	3O Not applicable
F.2.5	Employer(s)	1O yes	2O no	3O Not applicable
F.2.6	Religious leader	1O yes	2O no	3O Not applicable
F.2.7	Public	1O yes	2O no	3O Not applicable
F.2.8	Other (specify)	1O yes	2O no	3O Not applicable
F.3	How many people know about your HIV status in your home where you usually live? Would you say that it is no one, a few of the people, half of the people, most of the people, or everyone?	1O No one<br>2O A few of the people<br>3O Half of the people<br>4O Most of the people<br>5O Everyone		
